# 844 Modified Below-knee Amputation with Medial Gastrocnemius Muscle Flap and RPNI

**DOI:** 10.1093/jbcr/iraf019.375

**Published:** 2025-04-01

**Authors:** Mare Kaulakis, Christopher Fedor, José Arellano, Hilary Liu, Jenny Ziembicki, Francesco Egro

**Affiliations:** University of Pittsburgh School of Medicine; University of Pittsburgh School of Medicine; University of Pittsburgh Medical Center; University of Pittsburgh Medical Center; University of Pittsburgh Medical Center; University of Pittsburgh Medical Center

## Abstract

**Introduction:**

Below-knee amputation (BKA) may be required for patients with full-thickness burns to the lower extremities when the muscle or bone is deemed non-salvageable. Traditional BKA techniques can result in complications such as inadequate soft tissue coverage and phantom limb pain.

**Methods:**

This case report presents a modified BKA technique incorporating muscle flaps and Regenerative Peripheral Nerve Interfaces (RPNIs) to provide additional padding and mitigate postoperative pain.

**Results:**

We report on a 61-year-old male who sustained full-thickness burns with 65% Total Body Surface Area (TBSA) due to a flame injury. Initial management included escharotomies and the application of cadaveric allografts to the lower extremities.

Approximately three weeks post-admission, extensive necrosis and bone/tendon exposure necessitated bilateral BKA. The procedure was modified by transposing bilateral medial gastrocnemius flaps to provide enhanced padding for prosthetic use and a vascularized bed for future skin grafts. Additionally, RPNIs were performed by suturing three free muscle grafts around the dissected nerve endings to reduce postoperative pain. RPNIs were performed on the left stump using the superficial peroneal and tibial nerves, while the right stump received a tibial nerve RPNI.

Postoperative care involved posterior knee splints to prevent contractures. The patient developed left lower extremity phantom limb pain one week after surgery, which was alleviated by a sciatic nerve block and gabapentin.

Seven months post-amputation, the patient was fitted for prosthetics and began rehabilitation. Although intermittent phantom limb pain persisted, it was managed effectively with a regimen of over-the-counter pain medication. One year later, the patient had made significant progress in mobility and pain control, with continued efforts toward full-time prosthetic use.

**Conclusions:**

This case demonstrates the successful use of bilateral medial gastrocnemius flaps and RPNIs in a patient undergoing bilateral BKA due to burn injury. This innovative approach suggests a potential new standard for managing complex lower limb amputations in burn patients, offering enhanced soft tissue coverage and reduced postoperative pain.

**Applicability of Research to Practice:**

The modified BKA technique described in this case could be considered for broader application in burn patients requiring lower limb amputations. The dual benefits of improved tissue coverage and pain management may lead to better patient outcomes and quality of life.

**Funding for the Study:**

N/A

